# Link between Food Energy Density and Body Weight Changes in Obese Adults

**DOI:** 10.3390/nu8040229

**Published:** 2016-04-20

**Authors:** Marta Stelmach-Mardas, Tomasz Rodacki, Justyna Dobrowolska-Iwanek, Anna Brzozowska, Jarosław Walkowiak, Agnieszka Wojtanowska-Krosniak, Paweł Zagrodzki, Angela Bechthold, Marcin Mardas, Heiner Boeing

**Affiliations:** 1 Department of Epidemiology, German Institute of Human Nutrition Potsdam-Rehbrücke, Nuthetal 14558, Germany; boeing@dife.de; 2 Department of Pediatric Gastroenterology and Metabolic Diseases, Poznan University of Medical Sciences, Poznań 60-572, Poland; jarwalk@ump.edu.pl; 3 Department of Food Chemistry and Nutrition, Medical Collage Jagiellonian University, Kraków 30-688, Poland; rodak13@wp.pl (T.R.); justyna.dobrowolska-iwanek@uj.edu.pl (J.D.-I.); mfkrosni@cyf-kr.edu.pl (A.W.-K.); Pawel.Zagrodzki@ifj.edu.pl (P.Z.); 4 Department of Human Nutrition, Warsaw University of Life Sciences-SGGW, Warszawa 02-776, Poland; anna_brzozowska@sggw.pl; 5 German Nutrition Society, Bonn 53175, Germany; bechthold@dge.de; 6 Department of Human Nutrition and Hygiene, Poznan University of Life Sciences, Poznań 60-624, Poland; mmardas@up.poznan.pl

**Keywords:** body weight, energy intake, obesity, adults

## Abstract

Regulating the energy density of food could be used as a novel approach for successful body weight reduction in clinical practice. The aim of this study was to conduct a systemic review of the literature on the relationship between food energy density and body weight changes in obese adults to obtain solid evidence supporting this approach. The search process was based on the selection of publications in the English language listed in public databases. A meta-analysis was performed to combine individual study results. Thirteen experimental and observational studies were identified and included in the final analysis. The analyzed populations consist of 3628 individuals aged 18 to 66 years. The studies varied greatly in terms of study populations, study design and applied dietary approaches. The meta-analysis revealed a significant association between low energy density foods and body weight reduction, *i.e.*, −0.53 kg when low energy density foods were eaten (95% CI: −0.88, −0.19). In conclusions, this study adds evidence which supports the energy density of food as a simple but effective measure to manage weight in the obese with the aim of weight reduction.

## 1. Introduction

The perception and understanding of the energy density of foods is an important issue concerning dietary intake and the creation of more sustainable consumption patterns [1]. Food energy density is defined as the energy content (in kcal or kJ) per unit of weight (g or 100 g) [2]. Recently, food energy density has been recognized as an important factor which may significantly influence energy intake. Most natural foods of plant origin exhibit low energy density with exceptions of plant oils and nuts. Further, most of the low energy dense foods are characterized by high water and fiber content relative to high energy dense foods [2]. Another component of food which effects energy density is the fat content (9 kcal/g) which increases the energy density of a food to a greater extent than either carbohydrates (4 kcal/g) or proteins (4 kcal/g) [3]. It has previously been shown that consumption of foods with a high energy density increases energy intake in contrast to foods with low energy density (*i.e.*, fruits, vegetables, whole grains). Nevertheless, the real effect of fat content can be investigated by manipulating the ratio of fat to carbohydrates in diets while maintaining a constant energy density, or by manipulating the energy density of diets while keeping macronutrient content and palatability constant [4,5,6,7].

The prevalence of overweight and obese people is on the rise [8] and it appears that a diet able to self-regulate dietary energy intake may play a prominent role for obese individuals in the strategy of successful long-term weight loss, in the maintenance of weight, and also in the prevention of body weight gain [9,10]. Thus, the incorporation of low energy dense foods into the habitual diet of already obese individuals could be a crucial element for reducing energy intake and practicing successful body weight management. Thus, the concept of regulation food energy density could be a novel strategy for successful body weight reduction for practitioners and health professionals. Our objective was to update and summarize the information from the past ten years originating since the landmark paper published by Drewnowski *et al.* [11]. It has been shown that in crossover laboratory studies, energy-dense diets were associated with higher energy intakes and, in some cases, modest weight gain. However, cross-sectional studies were incapable of demonstrating causal associations between dietary energy density and weight change [11].

We therefore conducted a systematic review in order to study whether the evidence for an association between the energy density of foods and body weight changes in obese adults is as consistent and important in quantity as previously outlined.

## 2. Experimental Section

### 2.1. Search Strategy

Between February and December 2015 we have systematically searched the databases MEDLINE, SCOPUS, THE COCHRANE LIBRARY, WEB OF SCIENCE AND EMBASE to identify experimental and observational studies describing the energy density of food consumed in relation to body weight changes. The search strategy was restricted to obese adult human populations, and to articles written in English. It included the following types of documents: articles, reviews, books, and book chapters. Articles published in the last 10 years were analyzed. The search was based upon the following index terms contained within the title or abstract: #1 “food and beverages” or “energy density” or “caloric density” or “energy dense”; #2 “body weight or body weight changes”; #3 “obesity or obesity, morbid or obesity, abdominal”. Search #1 and #2 and #3.

The protocol was registered in “PROSPERO International prospective register of systematic reviews” PROSPERO 2014: CRD42014014007 [12]. The manuscript followed the PRISMA Statement on how to conduct a systematic review [13].

### 2.2. Inclusion and Exclusion Criteria

Those studies conducted in obese, adult populations and indicating food energy density in relation to body weight changes were eligible for this systematic review and those which used Food Frequency Questionnaire (FFQ), food records or 24-h dietary recalls as methods for assessment of dietary intake were analyzed.

Studies published in the last 10 years with the following design characteristics were considered: experimental (intervention studies), randomized controlled trials, observational (individual), cohort study, case-control study, cross-sectional study (surveys) and ecologic studies (population). We excluded publications which did not meet the inclusion criteria such as studies performed in specific groups of patients or other age-specific groups, animal studies, papers without sufficient dietary data to be able to make a conversion to energy density, other than above mentioned type of documents, and articles in any other language than English.

### 2.3. Data Extraction and Analysis

The study selection process was subdivided into a. titles, b. abstracts and c. full texts, and was performed by two independent researchers in parallel with each database. In each step all disagreements between the researchers was resolved after consultation with the review coordinator. Only in the case of agreement a document passed to the next round. Full-texts of all studies were obtained through libraries. In special cases, the corresponding or leading author was contacted via e-mail. For each full text paper, information was extracted according to general information (study title, authors, year, journal), study characteristics (study design, country, inclusion and exclusion criteria, length of intervention/observation or follow-up), characteristics of studied population (number, ethnicity, demographic characteristics of participants), assessment methods (body weight measurement, FFQ, 24 h dietary recalls, food records), physical activity and type of outcome (body weight changes).

The relationships between energy density of food and body weight changes were described as “favors low energy density” if lower food energy density was linked with reduction of body weight, and “favor high energy density” if lower food energy density was linked with increase of body weight or if there was no change in body weight. A 9-point scoring system according to the Newcastle-Ottawa Scale was used to assess the study quality [14]. A high-quality study was defined by a threshold of ≥7 points.

### 2.4. Statistical Approach

Where possible, the recorded energy intake levels were converted to kilocalories (kcal) and energy density of food into kilocalories per gram (kcal/g) in order to standardize results. A meta-analysis was performed to combine the results of individual studies. Data were analyzed using a random-effects model which allowed for the true effect to vary from study to study. The effect size of a study was investigated by calculating the standardized mean difference with a 95% confidence interval. The heterogeneity of the sum of studies was tested for significance. As a measure for quantifying inconsistency, *I*^2^ was selected [15]. Although included studies in our analysis were heterogeneous, carefully inclusion of the suited arms (similar approach for the intervention based on the diet composition) in different interventions allowed us to combine the collected papers and run an analyses still capable of separately identifying significant effects observed for either study design: RCTs or cohort studies. The results of the meta-analysis were visualized using a forest plot which illustrates the results of the individual studies and the summary effect. The analysis was performed with Review Manager (RevMan) V5.3 (The Nordic Cochrane Centre, the Cochrane Collaboration, Copenhagen, Denmark, 2014).

## 3. Results

### 3.1. Search Results

**Figure 1:**
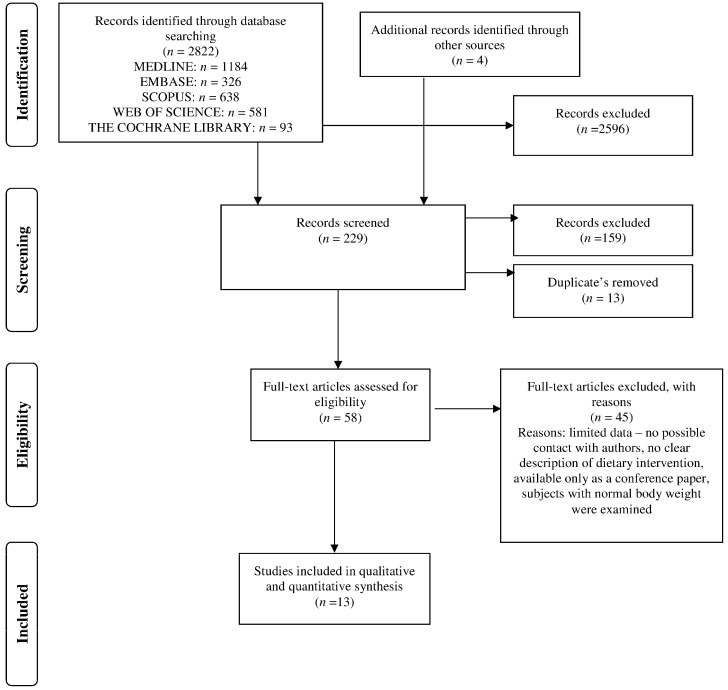
Process of literature search on the association between food energy density and body weight changes in obese adults.

The search process is presented in Figure 1. We identified 2822 potentially relevant publications and included 229 articles according to title and 58 according to abstract in the full text review. Finally, after removal of duplicates with multiple publications of the same study and sufficiency of data given in the publications, thirteen studies met the inclusion criteria and form the basis for generating the evidence for the described meta-analysis.

### 3.2. Study and Population Characteristic

Baseline characteristics of the thirteen studies and populations (*n* = 3628) are presented in Table 1. The number of individuals analyzed in each study ranged from 32 to 771. The age of subjects ranged from 18 to 66 years.

Six studies [16,17,18,19,20,21] were performed only in a female population, and in most of the other studies a higher percentage of women was reported. The studied populations covered American and European nationalities. The studies were designed as randomized controlled clinical trials (*n* = 9) or cohort studies (*n* = 4). The duration of the studies varied from 8-weeks to 9-months in interventions based on energy density of foods and up to 6-years of follow-up in observational studies [16,17,18,19,20,21,22,23,24,25,26,27,28].

### 3.3. Body Weight Changes in Relation to Energy Density of Foods and Energy Intake

**Figure 2:**
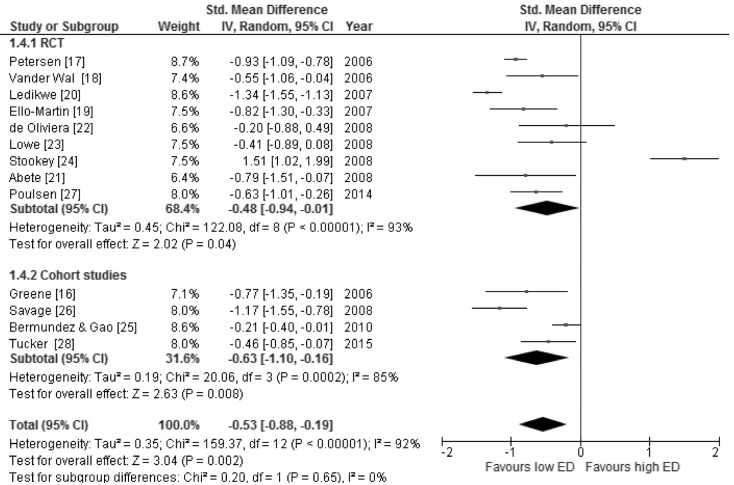
Forest plot of the random-effects meta-analysis of changes in body weight according to food energy density shown as polled standard differences in the means with 95% CIs and in selected cross-sectional studies and randomized trials. For each study, the square represents the point estimate of the intervention effect. Horizontal lines join the lower and upper limits of the 95% CI of this effect. The area of shaded squares reflects the relative weight of the study in the meta-analysis. Diamonds represent the subgroup mean difference and pooled mean differences.

**Figure 3:**
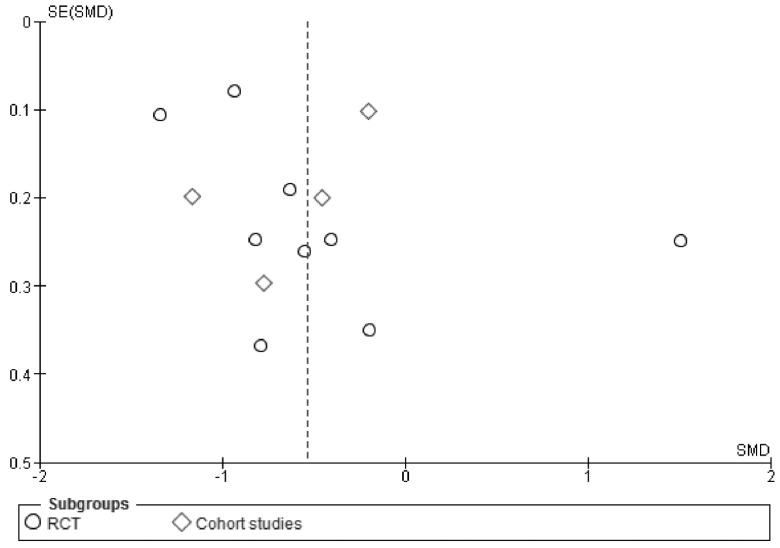
Funnel plot of standard error by standard differences in means of body weight.

The dietary strategies leading to a putative decrease in food energy density were based either on reduced fat content in the diet or a lower sugar intake [16,17,18,19,20,21,22,23,24,25,26,27,28]. However, details about the energy density of foods, at the baseline and at the end of the intervention, were reported only in six studies [16,17,19,22,25,28]. The mean body weight at baseline ranged between 77.3 ± 10.8 kg and 110.9 ± 14.7 kg. After the intervention period, the mean body weight decreased in most studies and was mainly associated with lower energy intake [16,17,18,19,20,21,22,23,24,25,26,27,28]. Energy expenditure of study participants was analyzed in two studies [23,26] and did not differ significantly during the intervention period (Table 2). The quantitative meta-analysis revealed a significant association between change in body weight (−0.53 kg; 95% CI: −0.88, −0.19) and the energy density of foods (*p* = 0.002, *I*^2^ = 92%) (Figure 2). In sub-group analyses regarding design, cohort studies (−0.63 kg; 95% CI: −1.1, −0.16; *p* = 0.008, *I*^2^ = 85%) and RCTs (−0.48 kg; 95% CI: −0.94, −0.01; *p* = 0.04, *I*^2^ = 93%) obtained similar results. The funnel plot did not reveal asymmetry despite 3 studies being outliers, suggesting no real evidence of a publication bias (Figure 3).

## 4. Discussion

These data show that a significant link exists between foods with low energy density and a decrease in body weight in obese subjects. The RCTs and cohort studies provide consistent evidence that the consumption of low energy density foods is associated with body weight loss in obese adults.

The results of the current systematic review are similar to a published systematic review on this topic which concluded that a dietary pattern of low energy density foods improves weight loss and favors weight maintenance [29]. However, authors pooled data from overweight and obese adults and children and did not perform a meta-analysis [29]. The results of RCT studies were not consistent in terms of body weight reduction. However, the data from non-controlled trials showed that, after approximately 10.5 months, energy density was reduced by 0.34 kcal/g from 1.58 ± 0.40 to 1.24 ± 0.38 kcal/g (*p* < 0.001) [21]. Evidence from the prospective cohort studies documented a positive association between lower energy density and decreased weight gain or BMI, better weight maintenance and/or weight loss and lower increases in waist circumference [29]. To properly assess the relationship between food energy density and body weight changes we should take into consideration different calculation methods of food energy density that could either include all beverages or be restricted to those containing energy, or skip them in the calculation altogether [30,31,32]. Ello-Martin *et al.* [16] excluded caloric beverages (such as milk and juices) as well as non-caloric beverages from the calculation of food energy density, concluding that this might disproportionately influence the calculation of energy density due to the fact that beverages tend to be much lower in energy density than most foods. In contrast, Poulsen *et al.* [28] included beverages in the calculation, showing an approximately 0.5 kcal/g lower food energy density of analyzed diet as compared with results of Ello-Martin *et al.* [16]. Nevertheless, the final effect was similar showing the positive association between low energy dense foods and greater weight loss [16,17,28]. Johanson *et al.* [33] suggested that energy intake from drinks should also be characterized and used as a covariate in the analyses of energy density and risk of obesity. Based on the studies included in our review, a low-energy density diet can be characterized by a higher proportion of vegetables, fruits, whole grain products and a lower intake of fat [17,18,19,20,21,28].

The causal explanations of our finding related to well controlled laboratory studies [30,31] which showed that the weight of foods consumed over the day is more constant than the energy eaten. A low energy dense food has shown similar to higher satiety effects, similar levels of hunger, similar fullness after consumption and a reduction in energy intake compared to high energy dense foods [30,31]. However, Westerterp-Plantenga *et al.* [32] reported that obese women compared to non-obese women selected larger portions of foods than standard sizes with a higher energy density. In our meta-analysis, we analyzed, among other studies, those studies in which the dietary strategies based on addition of extra servings like “post-dinner snacks” [24] or extra fruits [17] to the habitual diet. Although such intervention did not show statistically significant reductions in body weight (de Oliveira *et al.* in Figure 2), the study suffered with no drop outs during the intervention period [17]. Drewnowski *et al.* [11] pointed out that the energy density of the diet had a larger and more robust impact on energy intake than any macronutrient, e.g., fat. A low-fat diet has lower energy density and might be higher in nutrient density when fat is replaced either by whole grain or vegetables. Such an effect might be minor when fat is replaced by sugar [16].

Impact of energy expenditure regarding our findings can also be excluded since a significant change in resting energy expenditure was not observed during the study period [23,26], although this would be expected when a hypo-caloric diet is applied. It was also postulated by Jéquier [34] in the 80’s that since basal metabolic rate (BMR) decreases with weight loss, the postprandial energy expenditure of some “post-obese” subjects can be lower than that of lean controls. However, it remains controversial whether the decrease of BMR during hypocaloric treatment of obesity is due to change in body composition, or if it represents a downregulation in cellular metabolism [35].

### 4.1. Limitations

Despite an increasing number of dietary intervention studies, the body of evidence remains limited by either study quality or small sample size. Only future clinical trials with long-term follow-up periods can address this limitation. Furthermore, some studies published in the grey literature may have been missed by our literature search. Visual inspection of funnel plot raises some concerns regarding a publication bias, which might originate from the fact that data collection covered different age-specific groups. The results show, however, the same direction of changes in energy intake across the lifespan. However, potential sex differences in food energy density should be considered. Martí-Henneberg *et al.* [36] showed that sex differences in energy intake and energy density are highly pronounced in adolescents. Nevertheless, in adulthood a significant trend towards decrease (*p* < 0.001 in both sexes) was also observed, but food amount decreased significantly only in females. Taking into account that population from included studies consisted mostly of females, it could be that the obtained effect was more prone to be significant. We believe that changing energy density towards lower values will result, in most cases, in a higher quality of the diet as recommended by World Health Organization [8].

### 4.2. Practical Application

Dietary approaches, based on energy density of foods, show strong evidence that it could reduce body weight and prevent weight regain. The arguments for such an approach come from experimental studies showing that the total amount of food is the driving force for satiety, and thus, the intake of low energy dense foods leads to a reduction in energy intake in obese subjects. In clinical practice it will be possible without high efforts to consult for a diet including high amounts of low energy dense foods that will result in a successful weight loss strategy. Recently the German Nutrition Society [2] highlighted the approach of energy density as a measure of weight management. However, practical consulting material is still missing to help practitioners applying this approach. In addition, simple to apply assessment tools of food energy density could be advantageous.

## 5. Conclusions

In conclusions, consumption of foods with low energy density is associated with a beneficial decrease of body weight in obese subjects.

## Figures and Tables

**Table 1:** Studies (*n* = 13) and population characteristic (*n* = 3628).

Study	Study Design	Number of Participants/Nationality/Gender	Length of Intervention; Number of Dropped out	Age (Year)	Assessment Methods	Anthropometry	Type of Exposure	New-Castle Ottawa Scale
Greene *et al.*, 2006 [22]	Cohort study	74/American/61 women	2 years; 4.05% dropouts	51.5 ± 12.9	4-day food record, anthropometrical measurements *	Body weight	Weight management and maintenance in relation to dietary pattern	8
Petersen *et al.*, 2006 [23]	RCT	771/Europeans/579 women	10-weeks; 15.8% dropouts: 13.6% (*n* = 53) in low-fat group and 18.3% (*n* = 70) in high-fat group	36–39	3-day weighted diet records before and during last week of study; 1-day weighted records in 2nd, 5th, 7th weeks, anthropometrical measurements *	Body weight and height, waist and hip circumferences, fat mass, fat-free mass	Low-fat diet: 20%–25% energy from fat, 15% from protein, 60%–65% from CHO; high-fat diet: 40%–45% energy from fat, 15% from protein, 40%–45% from CHO; both diets provided 600 kcal/day less than individually estimated energy requirement	8
Vander Wal *et al.*, 2006 [24]	RCT	80/Americans/61 women	8 weeks; 23.8% dropouts: 27.5% (*n* = 11) in PDS group and 20.0% (*n* = 8) in NS group	18–65	Questionnaire, anthropometrical measurements *	Body weight, BMI, waist circumference, fat%	Standardized bowl of ready-to-eat cereal (RTEC) 1. “post-dinner snack” (PDS): bowel of RTEC and 2/3 cup of low-fat milk after supper; 2. “no snack” (NS): no snacks after meals	8
Ello-Martin *et al.*, 2007 [16]	RCT	97/American/all women	1 year; 26.8% dropouts: 27.1% (*n* = 13) in RF + FV group and 26.5% (*n* = 13) in RF group	20–60	3-day diet records (2 weekdays and 1 weekend day) anthropometrical measurements *	Body weight, height, waist circumference	Reduction in fat intake in diet (RF) or reduction in fat intake + increased consumption of water-rich foods (RF + FV)	8
Ledikwe *et al.*, 2007 [25]	RCT	658/Americans/61% women	24-weeks	49.0–50.5	Two 24-h dietary recalls, anthropometrical measurements *	Body weight, height, waist circumference	Behavioral intervention	8
Abete *et al.*, 2008 [26]	RCT	32/Spanish/14 women	8 weeks; no dropouts	36 ± 7	3-day weighted diet records, anthropometrical measurements *	Body weight; waist and hip circumferences	higher-GI (84% of total carbohydrates from rice and potatoes), 2. lower-GI (84% of total carbohydrates from whole-meal pasta and legumes), both diet were energy restricted (−30% of individually measured total energy expenditure), and designed as: (% energy) carbohydrates 53%, proteins 17%, fat 30%	7
de Oliveira *et al.*, 2008 [17]	RCT (secondary data analysis)	49/Brazilian/all women	10 weeks; no dropouts	30–50	3-day diet records including 1 weekend day anthropometrical measurements *	Body weight, BMI, mid-arm circumference	Normal diet with addition of either three apples or three pears or three oat cookies	7
Lowe *et al.*, 2008 [18]	RCT	103/American/all women	14-weeks	43.9 ± 10.5	5-day food records, anthropometrical measurements *	Body weight, height	Cognitive-behavioral treatment (CBT); CBT with an enhanced food monitoring accuracy (EFMA) program; or these two interventions plus a reduced energy density eating (REDE) program	8
Savage *et al.*, 2008 [19]	Cohort study	186/American/all women	6-years	24.1–46.6	Three 24-h dietary recalls	Body weight, height	Food energy density: low, medium and high	7
Stookey *et al.*, 2008 [20]	RCT (secondary data analysis)	173/American/all women	12 months; 17.3% dropouts (*n* = 30; data on dietary intake not available) and 15.6% (*n* = 27)	25–50	Three 24-h dietary recalls at 4-time points anthropometrical measurements *	Body weight, height, waist circumference, % of body fat	Atkins, Zone, LEARN or Ornish diets	8
Bermudez and Gao, 2010 [27]	Cohort study	947/Americans/51.2% women	-	20–39	24-h Recall anthropometrical measurements *	Body weight and height	Sugar-sweetened beverages and added sugars in normal diet	7
Poulsen *et al.*, 2014 [28]	RCT	181/Danish/128 women	1 week run-in period, 26 weeks intervention; 18.8% dropouts (*n* = 34)	20–66	Dietary-compliance and satisfaction questionnaire; anthropometrical measurements *	Body weight, waist and hip circumferences	New Nordic Diet (NND) or average Danish diet (ADD)	7
Tucker *et al.*, 2015 [21]	Cohort study	228/American/all women	4 years; 25.4% dropouts (*n* = 58)	35–45	Questionnaire concerning soft drinks intake; 7-day weighed diet records; anthropometrical measurements *	Body weight	Habitual diets with soft drinks: sugar sweetened or artificially sweetened or no soft drinks	7

* multi-functional digital scale was used.

**Table 2:** Mean changes in body weight taking into consideration food energy density and energy intake in selected studies (results for completers).

Study	Analyzed Groups	Food Energy Density (kcal/g)	Energy Intake (kcal/Day)		Energy Expenditure kcal/Day	Body Weight (kg) Mean ± SD	
			Baseline	Intervention		Baseline	Intervention
Greene *et al.*, 2006 [22]	Two groups of men and women; Maintainers: *n*_1_ = 59 Gainers: *n*_2_ = 15	Maintainers: 1.58 Gainers: 2.01	Maintainers: 1608 Gainers: 1989	N/A	N/A	Maintainers: 87.7 ± 22.4 Gainers: 98.8 ± 33.9	Maintainers: 86.5 ± 22.7 Gainers: 106.8 ± 36.5
Petersen *et al.*, 2006 [23]	Two groups of women; HF *: *n*_1_ = 235, LF *: *n*_2_ = 251 Two groups of men; HF *: *n*_1_ = 77, LF *: *n*_2_ = 85	N/A * N/A	Women: 2029 ± 55 Men: 2675 ± 838 ^^^	Women HF: 1514 ± 258 LF: 1447 ± 258 Men HF: 1928 ± 312 LF: 1900 ± 442	Women HF: 1740 ± 226 ^#^ LF: 1744 ± 251 ^#^ Men HF: 2151 ± 323 ^#^ LF: 2119 ± 304 ^#^	Women HF: 97.4 ± 14.9 LF: 96.7 ± 15.2 Men HF: 110.9 ± 14.7 LF: 110.3 ± 17.6	Women HF: 91.3 ± N/A * LF: 90.0 ± N/A Men HF: 102.7 ± N/A LF: 102.7 ± N/A
Vander Wal *et al.*, 2006 [24]	Two groups of men and women; PDS *: *n*_1_ = 29 NS *: *n*_2_ = 32	N/A	PDS: 2316 ± 915 NS: 2383 ± 998	PDS: 2081 ± N/A NS: 1649 ± N/A	N/A	PDS: 109.97 ± 22.92 NS: 106.91 ± 15.87	PDS: 106.26 ± N/A NS: 102.20 ± N/A
Ello-Martin *et al.*, 2007 [16]	Two groups of women; RF *: *n*_1_ = 36 RF + FV *: *n*_2_ = 35	Baseline: RF: 1.85 ± 0.07 RF + FV: 1.74 ± 0.06; At the end: RF: 1.49 ± 0.07 RF + FV: 1.33 ± 0.04	RF: 1836 ± 68 RF + FV: 1937 ± 78	RF: 1307 ± 62 RF + FV: 1437 ± 60	N/A	RF: 90.2 ± 1.4 RF + FV: 90.8 ± 1.8	RF: 83.8 ± 1.7 RF + FV: 82.9 ± 2.0
Ledikwe *et al.*, 2007 [25]	Three groups of women and men: Advice group: *n*_1_ = 223 Established group: *n*_2_ = 219 Established + DASH group: *n*_3_ = 216	Advice group: 1.53 ± 0.03 Established Group: 1.69 ± 0.03 Established + DASH group: 2.11 ± 0.03	Advice group: 1596 ± 36 Established Group: 1720 ± 38 Established + DASH group: 1842 ± 42	Advice group: 1632 ± N/A Established Group: 1476 ± N/A Established + DASH group: 1396 ± N/A	N/A	Changes: Advice group: 1.1 ± 0.2 Established Group: 5.1 ± 0.4 Established + DASH group: 6.1 ± 0.4	
Abete *et al.*, 2008 [26]	Two groups of men and women; hGI *: *n*_1_ = 16 lGI *: *n*_2_ = 16	N/A	N/A	N/A	Baseline hGI: 1698 ± 245 lGI: 1621 ± 287 Interventionh GI: 1584 ± N/A lGI: 1522 ± N/A	hGI: 94.4 ± 13.1 lGI: 94.3 ± 16.1	hGI: 89.4 ± N/A lGI: 87.2 ± N/A
de Oliveira *et al.*, 2008 [17]	Three groups of women; A *: *n*_1_ = 13 P *: *n*_2_ = 13 C *: *n*_3_ = 7	Baseline: A: 1.67 ± 1.14 P: 1.72 ± 1.25 C: 2.20 ± 1.31 At the end: A: 1.64 ± N/A P: 1.65 ± N/A C: 2.06 ± N/A	A: 2401 ± 389 P: 2459 ± 464 C: 2383 ± 31	A: 2376 ± N/A P: 2439 ± N/A C: 2384 ± N/A	N/A	A: 77.25 ± 10.75 P: 79.41 ± 12.89 C: 78.74 ± 8.40	A: 75.93 ± 11.35 P: 77.24 ± 11.47 C: 78.01 ± 9.17
Lowe *et al.*, 2008 [18]	Three groups of women; CBT: *n*_1_ = 35 CBT and EFMA: *n*_2_ = 35 CBT, EFMA, and REDE: *n*_3_ = 33	N/A	2164 ± 631	1735 ± 417	N/A	Changes: CBT: −1.31 ± 4.71 CBT and EFMA: −0.32 ± 4.72 CBT, EFMA, and REDE: −2.22 ± 4.15	
Savage *et al.*, 2008 [19]	Three groups of women: Low ED: *n*_1_ = 61 Medium ED: *n*_2_ = 63 High ED: *n*_3_ = 59	Low ED: 1.3 ± 0.2 Medium ED: 1.7 ± 0.1 High ED: 2.1 ± 0.2	Low ED: 1514 ± 437 Medium ED: 1649 ± 394 High ED: 1737 ± 409	N/A	N/A	Changes: Low ED: 2.5 ± 6.8 High ED: 6.4 ± 6.5	
Stookey *et al.*, 2008 [20]	Four groups of women: At *: *n*_1_ = 42 Z *: *n*_2_ = 47 L *: *n*_3_ = 42 O *: *n*_4_ = 42	N/A	N/A	N/A	N/A	At: 86.5 ± 3.9 Z: 85.0 ± 2.7 L: 83.1 ± 2.6 O: 87.2 ± 3.2	At: 80.8 ± 5.2 Z: 82.5 ± 4.1 L: 81.0 ± 4.5 O: 85.2 ± 4.5
Bermudez and Gao, 2010 [27]	Q1 *: *n*_1_ = 184 Q4 *: *n*_2_ = 211	N/A	Q1: 2141 ± 812 Q4: 2626 ± 929		N/A	Q1: 77.8 ± 22.8 Q4: 84.0 ± 33.4	
Poulsen *et al.*, 2014 [28]	Two groups of women: NND: *n*_1_ = 86 ADD: *n*_2_ = 50	Baseline: NND: 1.10 ± 0.25 ADD: 1.15 ± 0.22 At the end: NND: 0.90 ± 0.24 ADD: 1.13 ± 0.27	NND: 2329 ± 462 ADD: 2447 ± 645	NND: 1965 ± 613 ADD: 2366 ± 675	N/A	NND: 89.7 ± 16.4 ADD: 90.3 ± 18.2	NND: 85.0 ± N/A ADD: 88.8 ± N/A
Tucker *et al.*, 2015 [21]	Three groups of women; S *: *n*_1_ = 44 Ar *: *n*_2_ = 46 NSD *: *n*_3_ = 61	N/A	2017 ± 324 (data not differentiated at baseline)	N/A	N/A	Changes: S *: 2.7 ± 5.1 Ar *: −1.0 ± 4.4 NSD *: −0.5 ± 5.1	

* Legend: HF—high fat diet group, LF—low fat diet group, N/A—not available, ^—habitual diet for all women and men enrolled in the study, respectively, #—resting metabolic rate, PDS—post-dinner snack group, NS—no snack group, RF—reduction in fat intake in diet group, RF + FV—reduction in fat intake + increased consumption of water-rich foods group, hGI—high glycemic index group, lGI—low glycemic index group, A—normal diet with addition of three apples group, P—normal diet with addition of three apples pears group, C—normal diet with addition of three oat cookies group, At—Atkins diet group, Z—Zone diet group, L—LEARN diet group, O—Ornish diet group, Q1—participant group in lowest quartile of servings of sweetened beverages, Q4—participant group in highest quartile of servings of sweetened beverages, Po—participant group eating habitual diet with extra serves of pork, B—participant group eating habitual diet with extra serves of beef, Ch—participant group eating habitual diet with extra serves of chicken, NND—New Nordic Diet group, ADD—average Danish diet group, S—participants consumed sugar-sweetened soft drinks, Ar—participants consumed artificially sweetened soft drinks, NSD—participants did not consume soft drinks. Reported number of participants refers to the individuals that completed the study.
